# Translation, cross-cultural adaptation and psychometric properties of the Urdu version of knee injury and osteoarthritis outcome score questionnaire for Pakistani population

**DOI:** 10.1186/s12891-021-04477-1

**Published:** 2021-06-26

**Authors:** Sahar Fatima, Syed Asadullah Arslan, Faiza Sharif, Ashfaq Ahmad, Syed Amir Gillani, Anna Zaheer

**Affiliations:** Faculty of Allied Health Sciences, University Institute of Physical Therapy, The University of Lahore, Lahore, Pakistan

**Keywords:** Pain, Validity, Reliability, Translations, KOOS, NPRS

## Abstract

**Background:**

Knee injury and osteoarthritis outcome score questionnaire is a widely used tool for measuring short and long-term patient-relevant outcomes following knee injury. KOOS is neither translated nor examined for psychometric properties before. Therefore, the aim of this study was to translate, culturally adapt and check the psychometric properties of the KOOS in Urdu.

**Methodology:**

The translation and cultural adaptation was performed according to pre-defined guidelines. A total of 117 participants (54 males and 63 females) were recruited. The study had two steps: 1) Translation and cultural adaptation 2) Reliability and validity testing. The reliability (test-retest and internal consistency at (95% confidence interval) as well as the validity (Convergent validity) of final Urdu version of KOOS was tested.

**Results:**

For all five domains, the KOOS Urdu version (KOOS-U) has demonstrated high test-retest reliability ICC = 0.90–0.96(CI = 95%). For all domains, the internal consistency was determined to be excellent (α = 0.82–0.96). There were no floor or ceiling impacts noted. Convergent validity was found to be good, as measured by Pearson’s correlation coefficient. The findings revealed a strong negative association between the KOOS-U (QOL and pain) and the NPRS. And there was a low to high positive correlation between five KOOS-U domains and all SF-12 domains, i.e., there was a significant positive correlation between the pain domains of both KOOS and SF-12 with the r = 0.87(*p* < 0.05).

**Conclusion:**

The Urdu version of KOOS is a valid, reliable, and responsive instrument to assess functional disability of patients with Knee Osteoarthritis with excellent psychometric properties.

## Background

Musculoskeletal disorders of the lower limb involve soft tissues, joints or muscles of the lower limb [1]. Knee osteoarthritis causing chronic knee pain is a frequent condition particularly in the older population aged between 50 and 60 years. Work potency is greatly decreased from the loss of knee joint function. Eventually, unemployment due to early and forced retirement are faced by people with knee pain [2].

Old age and enlarged weight instigate Osteoarthritis (OA) of knee that consequences in pain and debilitated muscles nearby the joint. Physical parameters could be handled by subjective Assessment or objective assessment. Weak muscles diminish aerobic ability and decrease forbearance for activities of everyday life, involving walking. Movement is the activity mainly described as a problem for those affected with knee OA [3]. The use of questionnaires as a method of data collection in health-care research both nationally and internationally has increased in recent years. The increasing emphasis on evidence-based health care makes it even more important that nurses understand the theoretical issues associated with such methods [4].

All the languages a country own play a significant role in the instructive advancements of the countries and they are as vital in Pakistan as in other countries. There are many languages in Pakistan such as Balochi, Punjabi, Pashtu, Sindhi, Saraiki, Urdu and so on but National Language of Pakistan is “Urdu”, and other languages are imitated as “Mother Languages”. Since Urdu is the national language so this was used in the current research to collect data identified with musculoskeletal issues [5].

The 42-item self-report questionnaire, KOOS has 5 reported dimensions consisting of, pain (9 items), other symptoms (7 items), function in daily living (17 items), function in sport and recreation (5 items), and knee-related quality of life (4 items). The scoring system of the KOOS utilizes a 5- point Likert scale, with anchors of zero (no problems) to 4 (extreme problems). This transformed score is calculated using the following formula: 100 − [(actual raw score × 100)/possible raw score range]. An aggregate score is not calculated from the KOOS, in fact, each dimension should be analyzed and interpreted separately. Short-form measurement includes Seven items from the function in daily living and function in sport and recreation subscales of the KOOS. This shortened format is known as the KOOS-Physical Function Short form (KOOS-PS). The KOOS-PS was developed using Rasch-based measurement methods [6]. Different measures including Cincinnati Knee rating system are used to measure knee pain [7] According to Matteo Carosi et al., transcultural reliability and validity of an Italian version of the constant murley score showed that, CMS-IT has good reliability and internal consistency [8]. In another study, carried out by Silvia Vigilianese et al., assessed the multidisciplinary rehabilitation outcome checklist in Italian validation of an instrument for risk of discharge in patients with total hip/knee replacement. This scale was used by 114 patients, ICC values for hip was 0.97 and for knee it was also 0.97. This study reveals that this scale has the ability to check the patient in a detailed manner at their discharge [9].

Another research was conducted by Maria Moutzouri et al., main purpose of this research was to assess the psychometric specialties of advanced Greek-style “KOOS or Knee Injury and Osteoarthritis Outcome Score” in patients with “TKR” i.e., total knee replacement. Participants before surgery medical condition and after surgery results at two instances, that is, during their discharge and 1 to 2 weeks post-surgery were assessed utilizing “Knee Injury and Osteoarthritis Outcome Score functioning in daily living”, and evaluation of “SF-12 Health”. Brilliant ‘internal accuracy,’ suitable ‘KOOS test-retest reliability,’ 5 KOOS sub-domains, independently ICC, 95% CI = 0.235–0.902 and 0.89, 95% CI = 0.843–0.927 has been developed. For construct validity priori hypothesis was established with “KOOS” count and subdomains for pain, symptoms and “Active Daily Living” associating fairly by KOOS-ADL [10]. Each and every individual can better understand and respond in her/his own mother or national language. Pakistan is a country with the vast diversity but the national language is Urdu. Most of the literate Pakistanis speak and write in Urdu. KOOS is in English and the Urdu speaking patients in Pakistan cannot understand the language of the questionnaire. Therefore, it is extremely needed to translate the English version of the KOOS into Urdu to get reliable and detailed information from patients of knee osteoarthritis.

## Methodology

This cross-sectional study was conducted over a period of almost 2 years and data were collected from(March 2019 to March 2020) The study was divided into 2 stages: Translation and cross-cultural adaptation or Psychometric testing of KOOS Urdu version. All methods were carried out in accordance with relevant guidelines and regulations [11]^.^

### Stage I: translation and cross-cultural adaptation process

#### Step I: initial translation

In adaptation, the first step was the forward translation. From the original language (the root language) to the target language, at least two forward translations were made of the instrument. The translations were contrasted in this manner, as it they recognize inconsistencies that might represent unclear terminology in the original language or discrepancies with how a term is interpreted. To achieve the best quality translation, the two translators belonged from different profiles or histories.

#### Step II: synthesis of these translations

A third, non-biased entity was added to the team to establish a combination of the two translations. This person’s function was to act as a mediator in the discussion of discrepancies in translation and to produce written evidence of the process. Working from the original questionnaire and the first version of the translator (T1) and the second version of the translator (T2), a combination of these translations. A written report was completed, meticulously describing the synthesis process, each problem discussed, and how it could have been solved. Instead of one party risking their emotions, it was crucial that all problems be settled by consensus.

#### Step III: Back-translation

The questionnaire was then converted back into the original language, working from the T-12 edition of the questionnaire and entirely oblivious to the original version. This was a validity testing method to make sure that the translated edition correctly represents the original version’s item information. The back translation process often magnifies unclear wording in the translations.

As with forward translations, the standard was assumed to be two back-translations. Two bilingual individuals with the source language (English) as their mother tongue have developed back-translations (BT1 andBT2). The two translators were not aware of the topics discussed nor knowledgeable of them, and ideally without medical history.

#### Step IV: expert committee

To obtain the cross-cultural equivalence of the interpreted instrument, the configuration of the Expert Committee was important. At least one methodologist, healthcare practitioner, language professional, as well as all translators (both forward and backward) and the translation synthesis recorder were included in minimum membership of the Expert Group. During this phase of the process, the original authors of the questionnaire were in close touch with the Expert Committee to answer questions and provide feedback.

The task of the Expert Committee was to compile all variants and components of the questionnaire, including the original instrument, instructions, scoring documents and all interpreted versions of the questionnaire (T1, T2, T12, BT1, BT2), and to create the pre-final version of the field test questionnaire. In finalizing the translated instrument, important decisions were reached by the expert committee and full written recording of the problems was made.

#### Step V: test of the pre-final version

Pre-test was the final step of the adaptation process. This field evaluation of the new questionnaire uses a pre-final version of subjects/patients from the goal environment, preferably between 30 and 40 participants. In this study the questionnaire was filled by 20 patients for pilot testing. The questionnaire is first completed by each subject and then questioned to explore what they think was intended by each questionnaire object and its answer. It discussed both the significance of the things and the answers. This means that the modified variant of an applied scenario still preserves its equivalence. To search for a high percentage of missed items or single answers, the distribution of responses was analyzed. The outcomes of this stage were compiled and forwarded to the AAOS Committee for approval with the other papers.

#### Step VI: submission of documentation to the AAOS Committee for appraisal

The final step in the adaptation process was the submission to the AAOS Committee of all the findings and formats. The Committee has confirmed that the recommended stages have been implemented and that the findings appear to be a fair reflection of this process [11].

### Stage II: psychometric testing

The total sample size was 117 which was calculated by Kline method [12], out of which 54 were male participants and 63 were female patients of knee osteoarthritis. The data was gathered after Institutional review board (IRB) approval from The University of Lahore teaching hospital, department of physical therapy. All procedures were carried out in compliance with the applicable rules and regulations. Before data assemblage the informed written consent was also taken from all the participants. The inclusion criteria were male and female of age range between 40 and 75 years [13], subjects with primary knee osteoarthritis diagnosed and referred from orthopedic surgeon, those who were able to read and speak the native Urdu language and willing to participate in the study, patients that were able to understand and complete self-report questionnaires. Patients and subjects who were excluded from the study were having limb length discrepancy, musculoskeletal deformity, severe inflammatory arthritis, patients who had history of intra- articular use of corticosteroids and those who were unable to understand Urdu language.

### Participants and testing

SF-12 Score, Numeric Pain Rating Scale (NPRS) for pain in addition to the Urdu version of Knee injury and Osteoarthritis Outcome score was completed by 117 patients with a variety of knee pathologies. The inclusion and exclusion criteria were the same as the pre-test stage.

### Reliability

The reliability of KOOS-U version was tested among 117 patients of Knee osteoarthritis. The patients were instructed to fill KOOS-U, NPRS, SF-12 (quality of life) [14] during their first visit. Other demographic features were also acknowledged. After 48 h, same patients without any treatment were ret-tested in the same manner by filling KOOS-U, NPRS (pain), SF-12 (quality of life). The test-re-test reliability was measured by evaluating intra-class correlation coefficient (ICC) at 95% confidence interval. The internal consistency of KOOS-U was assessed through Cronbach’s alpha values and item total correlation. Internal consistency is regarded acceptable when alpha value exceeds 0.60–0.80 or excellent when it is between 0.80–0.95 [15]. The item-total correlation was evaluated by Cronbach alpha.

### Validity

Convergent validity was assessed by determining correlation between KOOS-Urdu and SF-12 scale, Numeric Pain Rating Scale (NPRS. The r values are distributed as follows: *r* = 0–0.25, very low correlation; *r* = 0.26–0.49, low correlation; *r* = 0.5–0.69, moderate correlation; *r* = 0.7–0.89, high or strong correlation; *r* = 0.9–1.0, very high or very strong correlation [16].

### Data analysis

Quantitative variables, in terms of mean ± SD and qualitative variables as frequency and percentage were taken to analyze the result by using SPSS version 23. Reliability was measured by test-retest reliability across repeated measures, internal consistency and measurement error by using an intra-class correlation coefficient (ICC2,1) and 95% confidence intervals (CIs). *P* < 0.05. Cronbach’s alpha was used to find Internal consistency.

## Results

### Translation and cultural adaptation

Urdu speaking 20 patients, both male and female with age range between 40 to 75 years of knee osteoarthritis were tested for face validity of pre final version of KOOS. To stay closer to the KOOS original version major changes were not made while translating it. Overall impression about Urdu version of KOOS was that it was easy to comprehend and feasible to complete all questions in 5 domains of this questionnaire. All items and their respective questions were relevant to concept it measuring. Therefore, no major changes were made in pre-test results of Urdu version of KOOS.

#### Reliability testing

In this study 117 patients having knee osteoarthritis were included. The mean age of patients was 53.68 years. There were 54(46.20%) males and 63(53.80%) females. The demographic details were presented in Table 1. The reliability statistics of all five domains is summarized in Table 2. The KOOS-U has shown excellent test-retest reliability for all domains. As far as the internal consistency is concerned, it was found to be excellent as all of the results exceeded the recommended level (0.60) of Cronbach’s alpha. The values (0.60–0.80) were considered as appropriate and results above 0.80–0.95 were excellent [15]. Floor and ceiling effects of all domains were zero which is also indicative of good reliability except one domain i.e., sports = 3(2.6%).

**Table 1:** Descriptive Statistics for Age and Gender of Patients

**N**	117
**Male**	54
**Female**	63
**Mean**	53.68
**SD**	9.678
**Minimum**	40
**Maximum**	70

As presented in Table-I the male participants were 54 and female participants were 63, the mean was 53.68 and standard deviation was 9.6

**Table 2:** Test-Retest Reliability and Internal consistency for KOOS-U Domains (*n* = 117)

KOOS Domains	_**1**_st Measurement	_**2**_nd Measurement	ICC (95%CI)	Cronbach’s Alpha (***n*** = 117)
	Mean ± SD	Mean ± SD		
**Pain**	35.58 ± 8.98	34.52 ± 7.76	0.93 (0.90–0.95)	0.93
**Symptoms**	43.50 ± 11.15	43.69 ± 9.54	0.95 (0.93–0.96)	0.96
**ADLS**	39.11 ± 11.67	38.99 ± 11.03	0.99 (0.99–0.99)	0.99
**Sports**	25.90 ± 8.70	29.70 ± 8.64	0.96 (0.94–0.97)	0.96
**QOL**	36.19 ± 8.72	34.48 ± 8.63	0.90 (0.86–0.93)	0.90

#### Validity testing

Pearson’s correlation was used to test Validity of KOOS-U with NPRS pain and SF-12 scale as shown in Table 3 and Table 4. According to results, there was strong negative correlation between KOOS-U (QOL and pain) and NPRS. There was average correlation between five domains of KOOS-U with all domains of SF-12 i.e., strong positive correction between pain domain of both KOOS and SF-12 with the r = 0.87(*p* < 0.05).

**Table 3:** Convergent Validity Testing of KOOS-U (*n* = 117)

	r (Correlation)	***p***-value
**Pain Intensity vs. KOOS Pain**	−0.860	0.000
**Pain Intensity vs. KOOS** **Symptoms**	−0.505	0.000
**Pain Intensity vs. KOOS ADLS**	−0.55	0.000
**Pain Intensity vs. KOOS Sports**	−0.781	0.000
**Pain Intensity vs. KOOS QOL**	−0.35	0.000

**Table 4:** Convergent Validity Testing of KOOS-U (*n* = 117)

KOOS	GH	PF	RLPH	SF	RLEP	E/F	Pain	EW
**Pain**	0.17	0.46	0.38	0.42	−0.14	− 0.16	0.87	0.38
**Symptoms**	0.14	0.40	0.10	−0.06	−0.29	0.58	0.69	0.35
**ADLS**	0.18	0.56	0.66	−0.08	**−**0.10	− 0.00	0.50	0.17
**Sports**	0.11	0.57	0.56	0.25	−0.03	0.04	0.23	0.22
**QOL**	0.72	0.47	0.30	0.19	0.06	0.02	0.31	0.23

*GH* General Health, *PF* Physical Functioning, *RLPH* Role limitations due to physical health, *SF* Social Functioning, *RLEP* Role limitations due to emotional problems, *E/F* Energy/Fatigue, Pain, *EW* Emotional Wellbeing

## Discussion

The current study helped to differentiate cross-cultural translation of KOOS-Urdu along with its psychometric properties by using 9 distinct languages that are believed to be Polish [17], Japanese [18], French [19], Dutch [20], Portugese [21], Persian [22], Swedish [23], Arabic [24] and Chinese [25]. All of the data analyzed contained patients of Knee OA.

In the current study, KOOS-Urdu was used among patients with knee osteoarthritis. After 48 h, this tool was again used to check its validity. None of the floor or ceiling effect was observed. It was determined, that Urdu translated form of KOOS has decent reliability and cogency and it showed acceptable psychometric characteristics in patients with knee osteoarthritis. Urdu version of KOOS seemed to be simply understood and clear in apprehension to all populations. The study design of this study was “qualitative tool validation”.

Associated to populaces, the quantity of subjects along “Knee OA” is unusually great for Pakistan, directing as a chief influence on the “Public health care system”. Along the disorders demanding help in “ADL or Active daily living”, “Knee Osteoarthritis” is graded 2nd by the Labour and Welfare in Japan and National Livelihood Survey of the Ministry of Health. Along junction with aging people, there is a robust necessity for calculation equipment’s to evaluate the impression of “knee Osteoarthritis” specific for a previous “knee damage”, and assess results from the subject viewpoint after particular treatments. Owing to the ongoing absence of obtainable Urdu participant self-evaluates instruments to assess “knee problems”, the purpose of the current study is to “multicultural adaptation” and evaluation of all the utmost extensively used patient self-rating knee outcome equipment’s, Knee injury and Osteoarthritis Outcome score. The “KOOS Urdu type” presented similar “consistency”, “interior consistency” and “construct validation” as compared to the initial Knee injury and Osteoarthritis Outcome score version and also further versions of languages [18].

The content validation was restrained by “index of content validity”. Content validation for relevancy, ambiguity, simplicity, and clarity ranged from 0.87–0.95. High Cronbach’s *α* coefficients were gathered for all sub-dimensions established that the KOOS subdomains were internally consistent and reliable with the items completely correlated with each other. Cronbach’s coefficients were at least 0.95 for almost all subdomains. The “convergence validity” was measured by “Pearson’s correlation coefficient”. Present study shows good results to demonstrate the validity and reliability of KOOS-Urdu. To the knowledge of the researcher, only research study that translated “KOOS” into “Urdu” and adjusted it “cross-culturally” was this study. The “psychometric properties” of “KOOS-Urdu” have been proved by a “pre-defined hypothesis”. The “adaptation process” showed that according to pre-established laws, “KOOS- Urdu” was efficiently translated. All the challenges encountered during the “adaptation process” were successfully resolved with the use of careful wording and compelling the majority conclusions of the “Expert review committee”. “KOOS-Urdu” is easy and convenient to use in clinical environments. The present study recruited 63 (“53.80%”) more females than 54 (“46.20%”) males, which is similar to previous surveys (“28 percent women”) [17]. Many researchers, however, have recruited more women than men. The “mean age” of patients in the current study was “53.68 years”, but patients with a slightly lower “mean age” were enrolled in another study (“36 years”) [26]. With the help of “Cronbach’s alpha (0.92)”, that is likewise in the context of findings from prior research, “internal consistency” was found to be outstanding in the current analysis (“0.75–0.99”) [20, 27]. The “item-total correlation” amongst a distinct piece and the entire count of “KOOS-Urdu” oscillated “0.82–0.92” which is somewhat close to the results of the “Chinese version of KOOS” (“0.89–0.95”) [28]. Excellent “test-retest reliability” ICC = 0.95 was established in the research that is analogous to the preceding studies of translation with excellent “test-retest” outcomes [24, 29]. However, the ICC value was found to be less in the Singapore (0.7) version of KOOS [25]. Because of the “interval variance” used to figure out the “test-retest reliability”, “test-retest” scores can differ. 48-h interval, similar to the preceding study was used in the current study with a lower “test-retest” interval, to confirm the least changes in patient status [21].

On the other hand, Mohammad H Ebrahimzadeh et al., recommended 72 h, to minimize the effects [30]. None of the “floor or ceiling effect” was discovered in the current research to get total values of “KOOS-Urdu” that is equivalent to the “Persian form” of “KOOS-Urdu” [31]. Instead, For the “KOOS Sports/Recreation” and “Quality of Life” scales, “Floor effects” only within “SDD” from the least count were found in another report. For the “KOOS Activities of Daily Living” “Ceiling effects” from the highest values inside the “SDD” were localized [32].

In the current study, KOOS-Urdu shows a positive correlation between KOOS-Urdu total score and SF-12 scale score which presented strong convergent validity. While, as compared to another study Maria Moutzouri et al., Receptiveness for Knee injury and Osteoarthritis Outcome Score sub dimensions of Symptoms and pain produced reasonable effect size that is ES = 0.4 [10]. In another study, MM Zulkifli et al., a large number of items were missing during the study. And there was also evidence that this study did not correlate KOOS with other tools. And because of this, there was a lack of any correlation, and no convergent validity was found [33]. On the other hand, the current study exhibits a strong correlation of KOOS between the “numeric pain rating tool” and “SF-12”. And no items were missing during this research.

### Limitations

- The first limitation was that, data was gathered only from patients of knee osteoarthritis and from physiotherapy Outdoor patient department only. Therefore, these results may not be applicable to check the quality of life of patients with other pathologies.
- As in this study, no treatment was provided to the patients therefore another limitation was that responsiveness (change over time) was not calculated.
- For test-retest reliability analysis, 48 h interval was considered. It could not be made sure that patient’s condition was kept unchanged.
- Moreover, due to this short interval the memory effects could not be controlled properly.

### Strengths

- The major strength of present study was that, the psychometric properties of KOOS-U were evaluated by means of pre-defined hypothesis.
- Another strength of this research study was that, the convergent validity was measured by using two different scales.

## Conclusion

- It is concluded that KOOS-U is reliable and valid questionnaire to assess level of pain in patients with knee osteoarthritis. It has simple and easy language that can be understood easily by the Urdu-speaking patients. Therefore, the clinicians and researchers should use KOOS-U to assess pain in Urdu-speaking patients having knee osteoarthritis.

## Data Availability

All data generated or analyzed during this study are included in this published article and its supplementary information files.

## Abbreviations

OA: Osteoarthritis
ICC: Intra-class correlation coefficient
NPRS: Numeric Pain rating scale
KOOS-U: Urdu version of Knee injury and osteoarthritis outcome score
KOOS: Knee injury and osteoarthritis outcome score
SEM: Standard error of measurement
SDC: Small detectable change
SF-12: Short form survey
